# Pharmacoeconomic evaluation of anti-obesity drugs for chronic weight management: a systematic review of literature

**DOI:** 10.3389/fendo.2023.1254398

**Published:** 2023-11-06

**Authors:** Yan Xue, Huimin Zou, Zhen Ruan, Xianwen Chen, Yunfeng Lai, Dongning Yao, Carolina Oi Lam Ung, Hao Hu

**Affiliations:** ^1^ State Key Laboratory of Quality Research in Chinese Medicine, Institute of Chinese Medical Sciences, University of Macau, Macao, Macao SAR, China; ^2^ School of Public Health and Management, Guangzhou University of Chinese Medicine, Guangzhou, China; ^3^ School of Pharmacy, Nanjing Medical University, Nanjing, Jiangsu, China; ^4^ Department of Public Health and Medicinal Administration, Faculty of Health Sciences, University of Macau, Macao, Macao SAR, China

**Keywords:** obesity, anti-obesity drugs, cost-effectiveness, modeling, methodology

## Abstract

**Introduction:**

Pharmacological therapy is recommended as a second-line alternative to reverse obesity. Currently, five anti-obesity drugs (AODs) have been approved by the U.S. Food and Drug Administration (FDA) for chronic weight management. The aim of this paper is to investigate the pharmacoeconomic evaluation of AODs through a systematic review with a special focus on methodological considerations.

**Methods:**

We searched the general and specific databases to identify the primary pharmacoeconomic evaluation of AODs.

**Results:**

A total of 18 full-text articles and three conference abstracts were included in this review. Most of the economic assessments were still about Orlistat. And the observations we could make were consistent with the previous systematic review. A few studies were on the combined therapies (i.e. PHEN/TPM ER and NB ER) compared to different comparators, which could hardly lead to a generalized summary of the cost-effectiveness. Most recently, pharmacoeconomic evidence on the newest GLP 1 RA approved for the indication of obesity or obesity with at least one comorbidity emerged gradually. Modelling-based cost-utility analysis is the major type of assessment method. In the modelling studies, a manageable number of the key health states and the state transitions were structured to capture the disease progression. In particular, the principal structure of the decision model adopted in the three studies on the newly approved drug was nearly the same, which enables more in-depth comparisons and generalizations of the findings.

**Conclusion:**

This study provided an up-to-date overview of the strengths and areas for improvement in the methodological design of the pharmacoeconomic evaluation of the licensed drugs for chronic weight management. Future modelling evaluations would benefit from a better understanding of the long-term weight loss effects of the current therapeutic options and the weight rebound process after the discontinuation of treatment.

**Systematic review registration:**

https://www.crd.york.ac.uk/prospero/display_record.php?ID=CRD42022302648, identifier CRD42022302648.

## 1 Introduction

The world has been experiencing an obesity crisis (1–4). According to the latest statistics of the World Health Organization, more than 1.9 billion adults (aged or older than 18 years) were overweight and around 650 million were obese. Between 1980 and 2015, a mounting prevalence of obesity was recorded at the global level (5). In the United States, more than 42% of adults were estimated to have obesity in 2018 (6). In China, the prevalence of obesity in adults was 16.4% from 2015 to 2019 according to recent national-wide nutrition surveys (7–9). The worldwide childhood and adolescent obesity issue is also worrying with consideration of its strong connection with adulthood obesity and other conditions in the long run (4, 10).

The elevated prevalence and incidence of obesity and overweight have been pressurizing the healthcare systems worldwide with complicated and serious health outcomes as well as multiplicatively unfavorable economic consequences. The linkage between obesity and overweight with increased occurrence of premature deaths, cardiovascular diseases, hypertension, type 2 diabetes, several types of cancers, as well as mental illnesses has been substantiated in various studies (5, 11–15). Besides the cosmetic concerns, undesirable health-related quality of life (HRQOL) has been consistently observed in the population with obesity (16–18). More recently, high-quality evidence was pooled to prove that the population group with obesity is vulnerable to COVID-19 in terms of incidence, morbidity and mortality, and is subject to compromised effectiveness of COVID-19 vaccines (19, 20). Financially, obesity and its related conditions lead to not only reduction and even loss of personal or family incomes, but also an increase in healthcare expenditure and other social costs (11, 21–23). Within the OECD countries, overweight and obesity were estimated to be responsible for 8% of their overall health budgets impacting 0.5%-1.6% of GDP (24).

Despite the profound implications of excessive weight, obesity remains an undertreated chronic disease and is often treated merely as a risk factor for other conditions (25–28). To reverse the trend of the obesity epidemic, both preventative and treatment interventions for weight normalization are needed (28–31). Life-style management has been prioritized for weight loss mainly by controlling energy intake from diets or boosting energy consumption with physical activities (32, 33). Bariatric surgeries are the recommended procedures for severe obesity with comorbidities owing to their proven effectiveness in sizeable weight reduction (34, 35). Pharmacological therapies are still categorized as a second-line auxiliary approach to treat obesity at designated obese stages or body mass index (BMI) levels with consideration of the occurrence of comorbidities (32, 33, 35–37).

The Food and Drug Administration (FDA) in the U.S. currently approves a handful of general anti-obesity drugs for long-term use, namely, orlistat, phentermine/topiramate extended-release (PHN/TPM ER), naltrexone/bupropion extended-release (NB ER), liraglutide (LIRA) 3.0 mg, and semaglutide (SEMA) 2.4 mg (38). In the latest network meta-analysis of the relevant randomized controlled trials, these pharmaceutical options could reduce 2.78 to 12.54% of the original weight (39) (please see details in **Supplementary Table S1**). Safety concerns pertaining to anti-obesity drugs (AODs), which are typified by high-profile market withdrawals due to severe adverse events of sibutramine, rimonabant, and the more recent lorcaserin, have led to more discretion in the approval of new drugs for weight loss purposes (40, 41). Orlistat (Xenical^®^) has been available on the market for more than 20 years and is the only one among the five long-term AODs approved by different major drug regulatory authorities including the U.S. FDA, the European Medicines Agency (EMEA), and the National Medical Products Administration (NMPA) in China. Notably, the recent discovery of novel treatment targets opened up new anticipated possibilities in pharmaceutical therapies for obesity with improved effectiveness and safety (42–44). In 2021, semaglutide 2.4 mg (Wegovy^®^) was approved to be on the American and European markets, which is the first drug authorized for chronic weight normalization since 2014 (38, 42, 45).

Cost-effectiveness evaluation is not only essential for pharmaceutical companies to prove the value for money of their innovative products to the regulatory authorities but also enables the manufacturers to predict the returns of their investment in a specific product (46). The pharmacoeconomic evidence on anti-obesity drugs has been emerging in several reviews which primarily focused on either pharmacologic treatment or various interventions (47, 48). Some of the drugs covered in those reviews have been de-licensed due to severe adverse events, e.g. sibutramine, rimonabant, lorcaserin while emerging studies on the cost-effectiveness of the two Glucagon-like peptide-1 Receptor Agonists (GLP-1 RAs) approved in 2014 and 2021 respectively have yet been included in any of the previous reviews. Therefore, it would be meaningful to pool the up-to-date relevant pharmacoeconomic studies together to obtain a more comprehensive overview of the currently available anti-obesity drugs for long-term use with a primary focus on the understanding of the pharmacoeconomic evaluation methods.

The aim of this paper is to investigate the published pharmacoeconomic evaluation of AODs through a systematic review with a special focus on methodological considerations. In particular, we aim to evaluate the model-based cost-effectiveness studies on their potential impact on the estimation of economic outcomes and discuss the possible structural uncertainty in the modelling approaches in the pharmacoeconomic evaluations of the drugs for chronic weight management.

## 2 Methods

The whole process of screening and selection of studies for inclusion according to the predefined eligibility criteria followed the Preferred Reporting Items for Systematic Reviews and Meta-Analyses (PRISMA) statement (49) (see **Supplementary Table S2**). The study protocol outlining the study design has been previously registered on the international prospective register of systematic reviews PROSPERO (reg. no. CRD42022302648).

### 2.1 Data sources and search strategy

The search for relevant studies was conducted in the mainstream electronic databases PubMed and EMBASE, as well as on the specific databases including ISPOR, Centre for Reviews and Dissemination (CRD) Databases (Database of Abstracts of Reviews of Effects (DARE), the National Health Service Economic Evaluation Databases (NHS EED), Health Technology Assessment Database (HTA). In addition, a snowball manual search was also performed by scanning the citation of eligible studies or relevant reviews. Both free texts and subject headings were adopted for searching the key concepts about obesity, anti-obesity drugs approved by the FDA for long-term use, as well as pharmacoeconomic evaluation. Zotero (5.0) and EndNote 9 (20.0 version) were employed for recording and managing the de-duplication and screening of articles retrieved from various sources, as well as reference management in writing the manuscript. We conducted the search on 23 January 2023 and no time limitation was set in the search. The language of studies was limited to English. See the **Supplementary Table S3** for the detailed search strategies used on different databases.

### 2.2 Eligibility criteria

Based on our study scope and aims, the eligibility criteria were predefined as outlined in **Table 1**. We primarily considered the original full pharmacoeconomic evaluations on any pharmacotherapy for chronic weight management currently approved by the FDA.

**Table 1:** Eligibility Criteria for Selecting the Included Studies.

	Inclusion criteria	Exclusion criteria
**Population**	- Obese/overweight patients with or without other comorbidities who have received pharmacotherapy for weight loss or maintenance	- Obese patients who have rare genetic diseases and need treatment with setmelanotide
**Intervention**	- Anti-obesity drugs approved by FDA for long-term use with or without lifestyle management	- Those drugs that have been withdrawn by the FDA by the inception of the current study - Setmelanotide
**Comparator**	- All the possible comparators in the relevant studies are considered, which may include other drug interventions, non-drug interventions, placebo, and no interventions	NA
**Outcomes**	- No restrictions are set on study outcomes. The potential relevant outcomes cover both measures of health outcomes (e.g. percentage of weight loss, kilograms of weight loss, changes in BMI, QALY, DALY, etc.) and economic outcomes (e.g. ICER, ACER, ICUR, etc.).	NA
**Study types**	- Full economic evaluations in which both the costs and outcomes are evaluated and compared with alternative interventions; - both data-based and modelling-based EE will be considered	- Partial economic evaluations that only report outcomes unrelated to costs, health outcomes, and/or economic evaluation outcomes - editorials, commentaries, reviews, theoretical papers, replies, viewpoints correspondences, and protocols

NA, not applicable.

### 2.3 Study selection

Based on the eligibility criteria, two reviewers first screened the titles and abstracts independently for initial inclusion. Then, full texts of articles considered eligible were reviewed by the two reviewers for the final inclusion. In both steps, reasons for exclusion were noted. And consensus between the two reviewers was reached over the final inclusion of studies by discussion.

### 2.4 Data extraction

An Excel form for data was designed and piloted by the main reviewer. The information to be extracted from the selected articles included the basic information of the study, the economic outcomes and conclusions on the cost-effectiveness, and the design of the pharmacoeconomic evaluations. The extraction of data was first performed by one reviewer, while the extracted data was later confirmed by another reviewer to ensure no omission or mistakes.

### 2.5 Quality assessment

The Consolidated Health Economic Evaluation Reporting Standards (CHEERS) Checklist (50) was employed for assessing the quality of the included studies on the 28 items. The full-text articles were evaluated against the 28 items with “yes” if they reported the relevant information and “no”, if not. The percentages of the studies reporting the items were calculated to obtain a general view of the completeness and quality of the studies.

## 3 Results

### 3.1 Selection of the included studies

**Figure 1:**
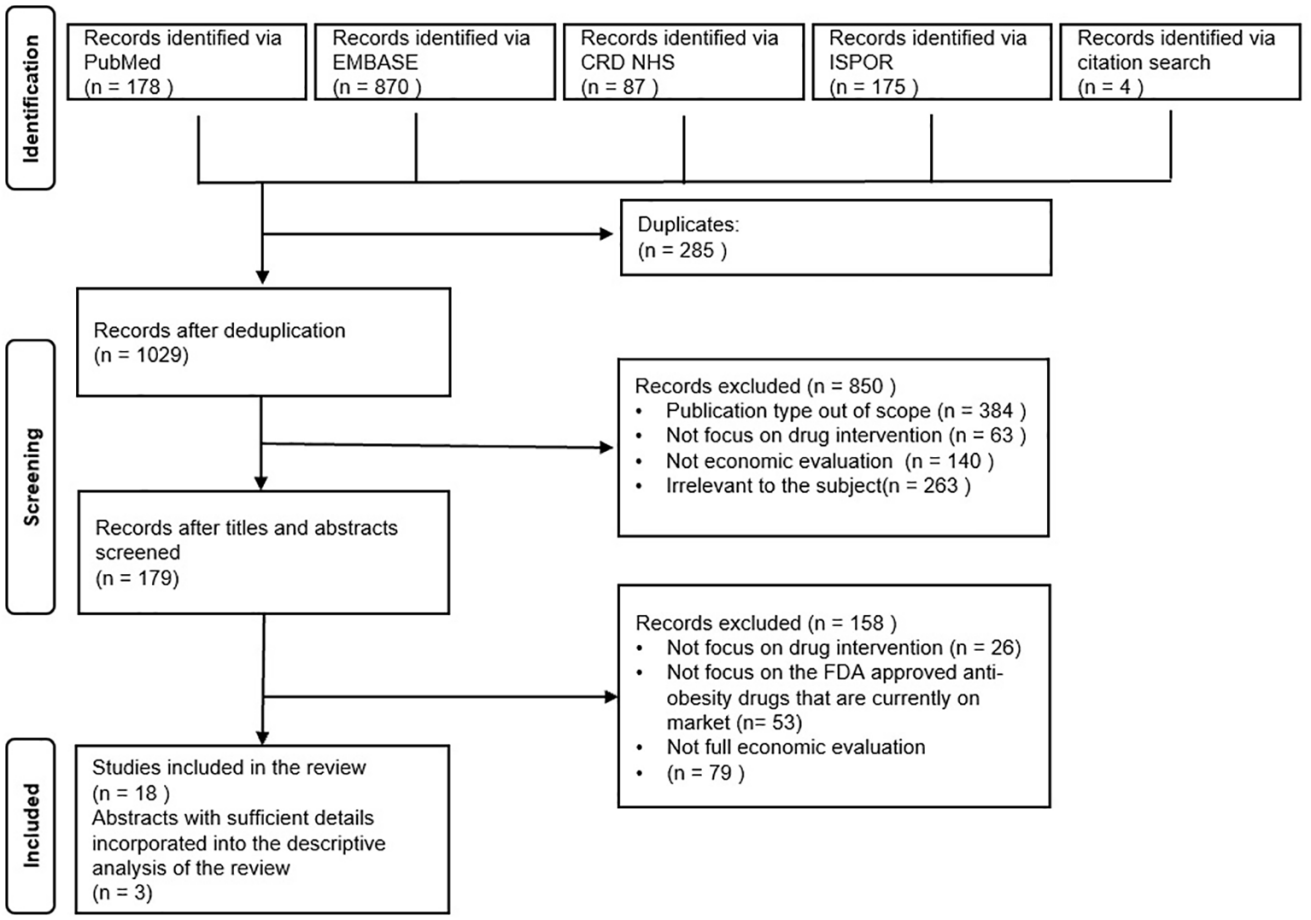
PRISMA flowchart of the study selection process.

A total of 1314 titles and abstracts were obtained initially for examination of their potential relevance to the current research focus based on the preset search strategy as illustrated in the previous section. After the removal of duplications, 1029 records were found valid for further screening. With a closer study of the titles and abstracts, a total of 179 records were identified as relevant to our research questions as outlined in the PRISMA Flowchart **Figure 1**. Thereafter, the full-text articles were retrieved and examined, and four additional research articles were found from the core references that fit with the eligibility criteria. Finally, 18 full-text published articles were included for the systematic review. Considering that only a limited number of primary pharmacoeconomic studies on some drugs could be searched, three conference abstracts with relatively sufficient information on their methodological design were also incorporated into the synthesis of information in the current study.

### 3.2 Quality assessment of the included studies

The quality of the 18 full-text articles included in the review was evaluated according to the CHEERS Checklist. The percentages of the studies reporting the 28 items were calculated and presented in **Supplementary Table S4**. All the included studies depicted their study context and settings, the objectives of conducting the economic evaluation, interventions or strategies for investigation, the baseline characteristics, and time horizon. Moreover, the measurement and estimation of health outcomes, resources, and costs were specified in all the full-text articles. However, the explanation of the reason for selecting a particular model structure and a very detailed description of the model were only seen in 2/3 of the studies. In the report of the results, the major study parameters and the main review findings were summarized. The effect of uncertainty was also included and discussed in all the studies. The limitations and generalizability of all the full-text studies were clarified. Notably, none of the studies have included any explicit efforts to engage patients or other stakeholders who are affected by the study, which is a new focus reflected in the latest version of the CHEERS checklist. All the studies in full text either reported their funding sources or disclosed conflicts of interest. Details of the quality assessment are presented in the **Supplementary Table S5**.

### 3.3 Descriptive characteristics of the included studies

The general characteristics of the included studies are presented in **Table 2**. Most of the studies were conducted in the UK and the European settings (51, 57, 58, 61, 63, 64, 66–69, 71), while 10 other studies were conducted in the US, Canada, or Australia (52–56, 59, 60, 62, 65, 70). One study analyzed the cost-effectiveness of AODs in more than one country (68). 14 studies examined the costs and benefits of orlistat as an adjunct intervention to either lifestyle interventions or dietary programs relative to other interventions, placebo or no treatment (55, 58, 60–71). The cost-effectiveness of phentermine/topiramate ER (PHN/TPM ER) was evaluated in five studies (52, 54, 55, 59, 60). Three studies examined naltrexone/bupropion ER (NB ER) (55, 56, 58), three examined liraglutide (LIRA) 3.0 mg (52, 55, 57). Regarding the latest approved semaglutide (SEMA), one study in 2020 examined semaglutide (SEMA) 0.4 mg (54), while the three most recent studies investigated the cost-effectiveness of the regimen in the approved dosage 2.4mg (51–53). Moreover, five studies performed comparisons between various approved AODs (52, 54, 55, 58, 60). The treatment duration either modelled or implemented in most studies normally lasted for around one year.

**Table 2:** General Characteristics of the Included Studies.

Author	Year	Ref No.	Country	Study type	Baseline condition	Intervention	Comparator	Duration of intervention/follow-ups	Funding source	Results		
										ICER/ACER (base case)	WTP threshold	Cost-effectiveness
Sandhu	2022	(51)	UK	modelling	a *post hoc* subgroup analysis of the STEP 1 trial: starting mean age was 48 years, and had an initial BMI of 38.7 kg/m^2^ having ≥ 1 weight-related comorbidity.	SEMA 2.4 mg weekly+ diet & exercise	diet & exercise alone	2 years	manufacturing company	GBP 14 827/QALY	GBP 20 000	cost-effective
Kim	2022	(52)	US	modelling	Step 1 trial data: average age 46 years old, BMI of 37.9 kg/m^2^	SEMA 2.4 mg weekly+ diet & exercise	LIRA 3.0mg	2 years	manufacturing company	USD 23 556/QALY	USD150,000/QALY	cost-effective
							PHN/TPM ER			USD 144 296/QALY		cost-effective
							NB ER			USD 127 518/QALY		cost-effective
							diet and exercise			USD 122 549/QALY		cost-effective
							no treatment			USD 27 113/QALY		cost-effective
Olivieri	2022	(53) (abstract)	Canada	modelling	average starting age and body mass index (BMI) were 50 years and 37.5kg/m2	SEMA 2.4 mg weekly	diet & exercise	1 years	manufacturing company	CAD31,861/QALY	CAD50,000/ QALY	cost-effective
Lee	2020	(54)	US	modelling	75% females and an initial age of 40 with initial BMI of 32.5 kg/m^2^	SEMA 0.4 mg daily	PHN/TPM ER	2 years	NR	1 year: USD 1 267 325/QALY	USD 100 000/QALY	not cost-effective
						SEMA 0.4 mg daily	ILI			3 years: USD 661 326/QALY		not cost-effective
						SEMA 0.4 mg daily	ILI			5 years: USD 520 262/QALY		not cost-effective
Finkelstein	2019	(55)	US	modelling	adults with BMI >25 kg/m^2^	PHN/TPM ER	weight watcher meetings	12 months	university fund	ICER: USD 501/additional kg lost in 12 months; ICER after 4 years: USD 117 219/QALY	USD 50 000/QALY	not cost-effective
						PHN/TPM ER	NA			ACER: USD 327 (245-422)/additional kg lost in 12 months, ACER (4 years): USD 75 137 (55 197-97 723)/QALY		NA
						NB ER	NA			ACER: USD 541 (389-689)/additional kg lost in 12 months, ACER (4 years): USD 122 451 (88 318-153 130)/QALY		NA
						orlistat	NA			ACER: USD 2028 (1 472-2 809)/additional kg lost in 12 months, ACER (4 years): USD 456 593 (315 955-657 942)/QALY		NA
						LIRA 3.0mg	NA			ACER: USD 2102 (1 548-2 648)/additional kg lost in 12 months, ACER (4 years): USD 479 177 (354 893-612 461)/QALY		NA
Nuijiten	2019	(56) (abstract)	Canada	modelling	adult patients who are obese or overweight in the presence of one or more weight-related comorbidities	NB ER	SM	NR	NR	ICER: USD 21 050/QALY	NR	dominant
Nuijiten	2017	(57) (abstract)	Switzerland	modelling	adults with obesity	Optifast LCD	LIRA 3mg	1 year		cost savings: CHF 9 732 (USD 10 437/EUR 9 633)	NR	meaningful costs savings
Fayter	2017	(58)	UK	modelling	adult patients who are obese or overweight in the presence of one or more weight-related comorbidities	NB ER+SM	SM	1 year	manufacturing company	ICER: GBP 13 647 (USD 17 753/EUR16 388)/QALY	GBP 20 000 (USD 26 018/EUR24 017)13/QALY	Cost-effective
						NB ER+SM	orlistat+SM			ICER: GBP 32 084 (USD 41 738/EUR 38 525)/QALY		not cost-effective
Finkelstein	2015	(59)	US	data-based	adult patients who are obese or overweight in the presence of one or more weight-related comorbidities	PHN/TPM ER (recommended dose 7.5/46 mg) + diet and exercise	Placebo + diet & lifestyle modification	56 weeks	university fund	ICER (taking Qsymia for 1 year with benefits linearly decaying over the subsequent 2 years): USD 48 340/QALY ICER (if benefits persist for only 1-year post drug cessation): USD 74 480/QALY	USD 50 000/QALY	may be cost-effective, depending on the time on Qsymia medication and whether QoL benefits persist 2 years beyond medication cessation
Finkelstein	2014	(60)	US	data-based	adults with obesity or overweight	orlistat	PHN/TPM ER (recommended dose 7.5/46 mg)	at least 1 year	manufacturing company	ACER per kilo of weight loss: USD 546 (390-736); ACER per QALY: USD 122 640 (88 530-164 440); ICER: dominated	USD 50 000/QALY	dominated
						PHN/TPM ER (recommended dose 7.5/46 mg)	Weight Watchers			ICER: USD 45 760-54 130		cost-effective
						PHN/TPM ER (recommended dose 7.5/46 mg)	NA			ACER per kilo of weight loss: USD 204 (134-317); ACER per QALY: USD 46,850 (32,010-69,350)		dominated
						orlistat	NA			ACER per kilo of weight loss: USD 546 (390-736); ACER per QALY: USD 122 640 (88 530-164 440)		dominated
Ara	2012	(61)	UK	modelling	average age of 45.5 years and a mean BIM of 34.92 kg/m^2^, 33.2% were diabetic	orlistat+diet and exercise advice	SM	12 months	NHS	ICER: GBP 1 665 (USD 2 166/EUR 2 000)/QALY	GBP 20 000 (USD 26 018/EUR24 017)	Cost-effective in the base case and different scenarios
Veerman	2011	(62)	Australia	modelling	obesity	orlistat	no intervention	1 year	government	ICER : AUD 230 000 (USD171 675/EUR158 482)/DALY (170 000-340 000)	AUD 50 000 (USD37 327/EUR34 406)	not cost-effective
Iannazzo	2008	(63)	Italy	modelling	adults with BMI >30	orlistat + lifestyle intervention	lifestyle intervention alone	4 years	manufacturing company	ICER for non-reimbursed (patients pay): EUR 75 000/QALY (8 000-181 000); ICER for non-reimbursed IGT group: EUR 21 000/QALY (-50 000-62 000); ICER for reimbursed: EUR 42 000/QALY (-22 000-109 000); ICER for reimbursed IGT: EUR 10 000/QALY (-60 000-39 000)	EUR 45 000/QALY	favorable for the IGT subgroup
van Baal	2008	(64)	The Netherlands	modelling	general population aged 20-70 years old with a BMI >=30 kg/m^2^, not treated for obesity before	orlistat + LCD	LCD only	1 year	government	ICER: EUR 59 000/QALY (19 000-59 000); ICER assuming direct relation between BMI and quality of life: EUR 24,100/QALY	NR	LCD recommended as the first option
Roux	2006	(65)	US	modelling	non-pregnant 35-year old women healthy, obese, or overweight without known co-morbidities	orlistat + diet	diet only	6-month intervention+6-month maintenance	government	NR	NR	weakly dominated
Foxcroft	2005	(66)	UK	modelling	adults with BMI 28-47 kg/m2	orlistat + diet	placebo + diet	1 year	manufacturing company	ICER with NICE criteria: GBP 24 431 (USD 31 780/EUR 29 337) (10 856-77 197); ICER with EMEA criteria: GBP 19 005 (USD 24 722/EUR 22 822) (8 440-57 798)	NR	NR; EMEA criteria recommended
Lacey	2005	(67)	Ireland	modelling	adults with BMI >=28 kg/m^2^, without diagnosed T2DM, the ability to lose 2.5 kg in weight during the introductory period.	orlistat + diet	placebo + diet	1 year	NR	ICER: EUR 17 000 (11 000 - 35 000)	NR	cost-effective if only treatment responders continue to use orlistat after three months
Ruof	2005	(68)	Sweden + Switzerland	modelling	overweight and obese patients with T2DM	orlistat + diet	placebo + diet	1 year	NR	ICER in Sweden: EUR 14 000 (80 000-21 000)/QALY; ICER in Switzerland: EUR 13 600 (7 000-21 000)	NR	NR; but supported the utilization and reimbursement of orlistat in relevant patient groups
Hertzman	2005	(69)	Sweden	modelling	BMI >=30 kg/m^2^, without T2DM, and be able to lose ≥2.5kg during 4 weeks before active treatment	orlistat + diet	placebo + diet	1 year	NR	ICER: EUR 13 125 (9 000 - 27 000)	NR	NR; comparable to an accepted healthcare treatment programme
Maetzel	2003	(70)	US	modelling	male patients with overweight or obese patients with T2DM at an average age of 52 years	orlistat+ATG+lifestyle modification	AGT + lifestyle modification	1 year	manufacturing company	ICER: USD 8 327/event-free LYG (6 791-25 827)	NR	cost-effective
Lamotte	2002	(71)	Belgium	modelling	obese type 2 diabetic patients without micro- or macrovascular complications	orlistat	No orlistat	2 years	manufacturing company	ICER with hypercholesterolemia+AHT: EUR 3 262/LYG; ICER free of events: EUR19 986/LYG	NR	cost-effective in obese diabetic patients (esp with hypercholesterolemia and/or hypertension)

ACER, average cost-effectiveness ratio; AGT, abnormal glucose tolerance; DALY, disability-adjusted life year; EMEA, European Medicines Agency; ER, extended-release; ICER, incremental cost-effectiveness ratio; IGT, impaired glucose tolerance; ILI, intensive lifestyle intervention; LCD, low-calorie diet; LIRA, Liraglutide; LYG, life-years gained; NB, naltrexone/bupropion; NR, not reported; PHN/TPM, phentermine/topiramate; QALY, quality-adjusted life year; SEMA, Semaglutide; SM, standard management; T2DM, type 2 diabetes mellitus; WTP, willingness-to-pay. NA, not applicable.

Most of the included studies are modelling-based (51–58, 61–71), which aimed to estimate the outcomes of weight reduction beyond the treatment duration by building mathematical models. And the baseline characteristics of the target population in these studies included the following categories: 1) obesity population with/without comorbidities, 2) overweight population with at least one obesity-related comorbidities, and 3) both conditions. Two studies exclusively focused on a gender-specific obesity group (65, 70).

All the studies with full-text research articles either disclosed the funding sources or the conflicts of interest, or both. Except for four funded by the government (61, 62, 64, 65), all the other studies involved the relevant pharmaceutical companies (e.g. Roche, Novo Nordisk, Vivus, etc.) in various forms.

Studies revealed that the general cost-effectiveness picture of the four anti-obesity drugs approved earlier for long-term use (i.e. orlistat, PHN/TPM ER, NB ER, LIRA 3.0mg) was not desirable. The cost-effectiveness of Orlistat varies largely in countries. For example, the model-based estimation of cost-effectiveness in the UK indicated that orlistat was cost-effective in the base case with an ICER of GBP 1 665 (USD 2 166/EUR 2 000) relative to placebo (61). However, in the Australian health care setting in 2003, orlistat was found to be not cost-effective with the ICER of AUD 230 000 (171 675 USD/158 482 EUR) per DALY (95% CI: 170 000 – 340 0000) in the base case in any of the costing scenarios (62). In addition, in the studies on the cost-effectiveness of orlistat, a range of cost-effectiveness thresholds was employed in the probabilistic sensitivity analysis to evaluate the impact of this threshold on the probability of cost-effectiveness of this intervention investigated. In terms of PHN/TPM ER, the ICER in a data-based CEA study turned out to be slightly below the WTP threshold of USD 50 000 per QALY, only if the benefit of the one-year treatment could be sustained for the following two years after drug cessation (59). And in another data-based study on PHN/TPM ER, the ICER was found to be at USD 54 130 per QALY and the average cost-effectiveness ratio (ACER) at USD 46 850 (32 010–69 350) per QALY with an assumed WTP threshold of USD 50 000 (60). In a more recent study, the ICER of PHN/TPM ER relative to a lifestyle management program called Weight Watcher was found to be as high as USD 117 219 per QALY (55). In the last two studies mentioned above, the ACERs of other pharmaceutical treatments including orlistat, NB ER, and LIRA 3.0mg were considerably higher than the commonly accepted WTP threshold of USD 50 000 (55, 60). Furthermore, the selection of different comparators led to different conclusions on the cost-effectiveness of NB ER. For instance, the two CEA studies of NB ER conducted in the health care setting of Canada and the UK respectively reported NB ER to be a cost-effective weight loss option relative to standard weight management for long-term use (56) and even in a lifetime horizon (58). However, in the later study, this combination therapy was found to be not cost-effective relative to orlistat (58).

Notably, the latest three studies on SEMA with the approved dosage at 2.4mg conducted in different settings converged on the conclusions about the cost-effectiveness of this newest anti-obesity drug approved by the major drug authorities. From the UK National Health Service (NHS) and Personal Social Services perspectives, the SEMA 2.4mg injection could benefit the population with obesity and relevant comorbidities with an ICER of GBP 14 827 per QALY relative to the treatment of diet and exercise alone (51). And a series of sensitivity analyses proved the robustness of its cost-effectiveness in different scenarios under the prespecified willingness-to-pay (WTP) threshold as GBP 20 000 per QALY. In a setting of US third-party payer, this newly approved therapy also showed its cost-effectiveness against all the selected comparators including three branded AOMs under the WTP threshold of USD 150 000 per QALY (52). In another assessment of the cost-effectiveness of SEMA 2.4mg injection in a Canadian setting, the therapy showed a favorable ICER at CAD31 861 per QALY when compared with diet and exercise under the WTP threshold suggested in the relevant Canadian Guidelines (CADTH) (53). However, in an earlier CEA study on SEMA 0.4mg administered per day from the US healthcare perspective, the ICER of the same therapy option given in a daily pattern with favorable weight loss effects was found to be not cost-effective in all the projected time horizons (54).

### 3.4 Analysis of the pharmacoeconomic evaluation methods

#### 3.4.1 Types of cost-effectiveness analysis

As summarized in **Table 3**, cost-utility analysis (CUA) is the major type of assessment method among all the included studies, with quality-adjusted life year (QALY) as a proxy of the health outcome (51–58, 61–64, 66–69). There was one study conducted in the Australian setting that used disability-adjusted life year (DALY) as a measure of health loss (62). A few studies undertook both CUA and cost-effectiveness analysis (CEA) with QALYs and kilograms of weight reduction as the measure of health outcome, respectively (59, 60, 65). In addition, two early studies only adopted event-free life years gained (LYG) as the measure of health benefit (70, 71). No cost-benefit analysis or cost-minimization analysis was observed in the economic evaluations of pharmacologic treatment for obesity.

**Table 3:** Details of the Included Pharmacoeconomic Evaluations.

Author	Year	Ref No.	Evaluation technique	Type of modeling approach	Time horizon	health states and clinical events modelled	Source of effect data	Source of health benefit	Study perspective	Cost categories	Discount rate (effect/cost)	Sensitivity analysis type	Model validation
Sandhu	2022	(51)	CUA	Markov (a UK adaptation of the Core Obesity Model)	40 years	Development of T2DS, first and second complications (including acute events e.g. knee replacement, bariatric surgeries), death	pivotal RCT (Step 1 trial)	prior literature	NHS and personal social services	obesity monitoring costs, health state costs, bariatric surgery costs, acute event costs, and AE treatment costs	3.5%/3.5%	DSA & PSA	Yes
Kim	2022	(52)	CUA	cohort Markov	30 years	treatment discontinuation; 5 mutually exclusive categories of health states: no comorbidity (ie, normal glucose tolerance or prediabetes), single comorbidity (ie, postacute coronary syndrome, T2D, poststroke, and cancer), dual comorbidity, multicomorbidity, and death; health events and acute complications considered in the model included bariatric surgery, acute coronary syndrome (myocardial infarction and angina), stroke (including transient ischemic attack), obstructive sleep apnea, and knee replacement.	pivotal RCTs (Step 1 trial)	prior literature	third-party payer	health care costs for obesity treatment, consultation, management of comorbidities, and obesity treatment-related adverse events (including bariatric surgeries as acute events)	3%/3%	DSA & PSA	Yes
Olivieri	2022	(53) (abstract)	CUA	Markov (Core Obesity Model)	40 years	NR	pivotal RCTs (Step 1&2 trials)	Prior literature	societal	NR	1.5%/1.5%	DSA & PSA	NR
Lee	2020	(54)	CUA	micro-simulation	1, 3 & 5 years	NR	clinical trials	prior literature	health care	cost of treatments, physician visits (first year), exclude costs of comorbidities and adverse events	3%/3%	DSA & PSA	No
Finkelstein	2019	(55)	CUA	NR	4 years	NR	Meta-analysis of RCR results	independent estimation	payer	direct medical costs, physician visit costs	3.5%/3.5%	DSA & PSA	No
Nuijiten	2019	(56) (abstract)	CEA	event-driven	20 years	NR	key clinical studies	NR	public health care payer	NR	NR	NA	NR
Nuijiten	2017	(57) (abstract)	CEA	event-driven	10years	NR	prior literature and data	prior literature and data	payer	NR	NR	NR	NR
Fayter	2017	(58)	CUA	discrete event simulation	lifetime	treatment discontinuation; development of T2DM; first and second CVD; death	pivotal RCTs	independent estimation	payer	drug acquisition costs, non-drug costs related to SM, comorbidity costs, and adverse event costs	3.5%/3.5%	DSA & PSA	Yes
Finkelstein	2015	(59)	CEA (CUA)	NR	3 years	NR	pivotal RCTs	independent estimation	payer	prescription cost, potential cost offsets from reducing medications for concomitant medications, physician appointment costs	3.5%/3.5%	DSA & PSA	No
Finkelstein	2014	(60)	CEA & CUA	NR	4 year	NR	Meta-analysis of RCT results	independent estimation	NR	weight loss medication and physician visit costs	NR	DSA & PSA	No
Ara	2012	(61)	CUA	cohort simulation model (i.e. Markov)	lifetime	time to death (ACM), primary MI, primary stroke and the onset of T2DM	clinical studies	independent estimation based on EQ-5D	NHS and personal social services	direct health-care costs	3.5%/3.5%	DSA & PSA	No
Veerman	2011	(62)	CUA	proportional multi-state life table Markov model	lifetime	colorectal cancer, breast cancer, endometrial cancer, kidney cancer, ischemic heart disease, stroke, hypertensive heart disease, type II diabetes, osteoarthritis	previous meta-analysis	independent estimation	health sector	costs of pharmaceuticals, GP visits, and lifetime health care costs (including health care expenditure for diseases related to obesity in later years)	3%/3%	DSA	No
Iannazzo	2008	(63)	CUA	probabilistic Markov model	10 years	diabetes onset, CVD, death	RCT results and prior literature	previous studies	societal	orlistat cost (by patient), direct medical cost of diabetes and obesity (by NHS)	3.5%/3.5%	PSA	No
van Baal	2008	(64)	CUA	(Markov) RIVM Chronis Disease Model	80 years	CHD, stroke, T2DM, osteoarthritis, low back pain, and cancer	RCT results and prior literature	independent estimation	health care	cost of orlistat and diet intervention, & dietitian visits	1.5%/4%	DSA &PSA	No
Roux	2006	(65)	CEA & CUA	first-order Monte Carlo simulation	lifetime	CHD risk profile (hypertension, type 2 diabetes, hypercholesterolemia), CHD, coronary death	clinical trials, population-based surveys, and published literature	primary study	societal	direct medical costs (obesity & obesity-related morbidity and mortality) & direct non-medical costs, participant time costs	3%/3%	DSA	No
Foxcroft	2005	(66)	CUA	Not stated	1 year	treatment responding	RCT results and prior literature	previous study	NR	prescription cost, GP visit costs	NA	DSA	No
Lacey	2005	(67)	CUA	Markov	11 years	T2DM, death	RCT results and prior literature	previous study	health-care	cost of orlistat, cost of the dietary programme, cost of diabetes monitoring and treatment health care costs	3%/3%	DSA	No
Ruof	2005	(68)	CUA	Markov model	11 years	diabetes-related micro/macrovascular complications, death	meta-analysis	previous literature	NR	NR	3%/3%	PSA	No
Hertzman	2005	(69)	CUA	Decision Tree- Monte Carlo	11 years	treatment responding; T2DM	previous RCTs	previous study	health-care	cost of orlistat, healthcare visits, costs of treating diabetes	3%/3%	PSA &DSA	No
Maetzel	2003	(70)	CEA	Markov model	11 years	diabetes-related microvascular or macrovascular complications	RCT results and prior literature and data	NR	health care	cost of orlistat, medical costs of treating other comorbidities	3%/3%	PSA	No
Lamotte	2002	(71)	CEA	Markov state transition model	10 years		RCT results and prior literature and data	NR	health care	cost of orlistat, medical costs of treating other comorbidities	0/3%	DSA	No

CEA, cost-effectiveness analysis; CHD, coronary heart disease; CUA, cost-utility analysis; CVD, cardiovascular diseases; DSA, deterministic sensitivity analysis; MI, myocardial infarction; NA, not applicable; NR, not reported; PSA, probabilistic sensitivity analysis; RCT, randomized controlled trial; T2DM, type 2 diabetes mellitus.

#### 3.4.2 Decision analytic approaches

Various decision- analytic approaches were observed in the modelling-based studies. Cohort-based Markov model was commonly applied to conceptualize a series of health states in relation to obesity and transitions between the states in most of the studies (51–53, 61–64, 67, 68, 70, 71). In particular, the latest publications on the SEMA 2.4mg adopted the Core Obesity Model with adaptations to various extents, which is indeed a typical Markov structure (51–53). The individual-based state-transition Monte Carlo simulation was also employed by modelling different patient characteristics with multiple runs in the model cycle representing the state changes in a few of the studies (54, 65, 69). In addition, the event-driven simulation was used in three studies to capture the complex disease course of obesity (56–58). The modelled health states or events include discontinuation of treatment, and occurrence of obesity-related events (e.g. type 2 diabetes, primary and secondary cardiovascular events, death). 10 of the studies provided a justification for selecting a particular model and a relatively detailed account of the decision model structure (51, 52, 58, 61–63, 65, 68, 70, 71). Moreover, the explicit model validation procedure was only mentioned briefly in two of the latest investigations on SEMA 2.4mg (51, 52).

#### 3.4.3 Perspective of the evaluation and cost categories

Most of the included studies specified their evaluation perspectives. The selection of cost categories also differs according to the study perspectives. Eight of the studies adopted a health-care perspective, involving the costs of the anti-obesity drugs, direct medical costs of treating obesity-related conditions, health care costs, and even the costs of the dietary programs (54, 56, 62, 64, 67, 69–71). The payer perspective was undertaken in eight of the studies, which mainly considered the costs of interventions and physician visit costs, and other medication costs for reducing obesity-related conditions (51, 52, 55–59, 61). Three studies performed their evaluation from a societal perspective (53, 63, 65).

#### 3.4.4 Time horizon projected and discounting

The two data-based studies focus on the outcomes within the one-year treatment period, no discounting was performed as unnecessary (59, 60). The modelling-based studies adopted various time horizons, among which five stretched the evaluation to a lifetime or around (58, 61, 62, 64, 65), three projected the outcomes in a period of 30 or 40 years (51–53), eight selected a time horizon between 10-20 years (56, 57, 63, 67–71), while the rest used a short-term time horizon no more than five years (54, 55, 66). Correspondingly, for the modelling-based evaluation with more than a one-year time horizon, discount rates that followed the guideline or consensus in a specific country or setting were applied to future effects and costs in most of the studies (51–55, 58, 59, 61–65, 67–71). Moreover, time horizon and discount rates were estimated at values different from the base case in the sensitivity analysis to investigate the parameter uncertainty in some of the studies (51, 52, 54, 58, 59, 61, 65, 67, 68, 70, 71).

#### 3.4.5 Sources of evidence and estimation of outcomes

Most of the studies reported the sources of data about effectiveness and health utility. The extrapolation of effectiveness (e.g. discontinuation data, rate of responders, mean change in body weight, risk of obesity-related sequelae, and adverse events) was mainly derived from the pivotal large-scale randomized control trials or meta-analysis (51–56, 58–71).

The valuing of health-related utility (i.e. QALY or DALY) in some of the studies was directly informed by published literature (51–54, 56, 57, 63, 65–69). Independent computation of health utilities was also found in several studies by transforming the effectiveness data into QALY or DALY with the aid of established algorithms (55, 58, 62, 64).

#### 3.4.6 Sensitivity and uncertainty analysis

Sensitivity analysis was carried out in all the studies in full text to check the robustness of the base case estimates. More than half of these studies performed both deterministic and probabilistic sensitivity analyses (51–55, 58–61, 64, 69). The covariates in the sensitivity analysis of these 18 studies fell into the following categories, namely, baseline characteristics, efficacy of interventions in comparison, natural weight increase rate, duration of weight loss benefit decay, occurrence of obesity-related conditions, costs and discount rates, valuation of health utility, and so on. However, there was no consistent inclusion of covariates among these studies. In addition, in many studies, authors solely listed specific variables or scenarios for analysis without giving detailed justification for selecting a specific parameter for the sensitivity analysis in advance. Among the evaluations on orlistat that were performed in the early 2000s, five of the studies only conducted a series of univariate sensitivity analyses by variating one of the input parameters each time (62, 65–67, 71), while the other three studies only performed probabilistic sensitivity analysis (PSA) with results displayed in scatter plots, cost-effectiveness/utility curves as well as planes as a measure of uncertainty (63, 68, 70).

## 4 Discussion

The current review comprehensively consolidated the pharmacoeconomic evidence relevant to drug options for long-term weight control. Different from the previous reviews on similar topics, the primary focus of this study rests on the methodological design of the pharmacoeconomic evaluations in the synthesis and analysis of the included studies.

Our predefined search strategy and selection process enabled us to access the relevant and up-to-date studies, of which the interventions cover all the five currently available anti-obesity drugs approved by the FDA. In general, these five AODs work on various peripheral and central pathways to regulate energy intake, suppress appetite, or increase fullness (72). Orlistat is an agent acting via peripheral pathway. It acts as an inhibitor of gastrointestinal and pancreatic lipase by preventing the catalysis of hydrolyzing triglycerides. Therefore, free fatty acids are not absorbed by the intestinal endothelium (45). Phentermine is a sympathomimetic amine anorectic acting as a norepinephrine agonist in the central nervous system, thus, decreasing the appetite. Its common anticonvulsant, topiramate, which is a gamma-aminobutyric acid agonist, glutamate antagonist and carbonic anhydrase inhibitor, shows several potential mechanisms of topiramate on weight loss (73). However, the clear mechanism of action of the combination therapy of PHN/TPM ER still awaits confirmation in animal and human studies (45, 74). NB ER is another combination therapy for long-term weight management that makes use of the synergistic effect of two distinct agents. Naltrexone originally is an opioid receptor antagonist, while bupropion a dopamine and norepinephrine reuptake inhibitor. In the hypothalamus, bupropion enhances the effects of pro-opiomelanocortin (POMC) cells in producing melanocyte-stimulating hormone (alpha-MSH) and beta-endorphin. The alpha-MSH activates melanocortin-4 receptor; which can decrease suppress appetite, and increase energy expenditure and weight loss. Naltrexone blocks mu-opioid receptor, so preventing the inhibitory feedback from beta-endorphin on POMC cells. Therefore, bupropion and naltrexone work complementarily to reduce bodyweight (45, 74). Lastly, both LIRA and SEMA are analogs of human glucagon like peptide (GLP-1) and act as GLP-1 receptor agonist. They stimulate pancreas to release insulin, which can regulate glucose concentration to reach euglycemia. They also inhibit the secretion of glucagon which triggers glycogenolysis and gluconeogenesis. In this approach, appetite and digestion are suppressed, thus calorie intake is reduced (45, 74, 75). Interestingly, the dose-dependent weight reduction effect of four among these five approved AODs (except Orlistat) was observed in the exploration of multiple sites of action and mechanisms of the therapeutic agent(s) involved, which were originally for other pathophysiological conditions (45, 75). This development process of these critical weight loss therapies benefits from the recent advances in the understanding of the pathophysiology of obesity as a complex disease and the metabolic processes (74).

The cost-effectiveness of the four AOMs before the approval of SEMA 2.4mg was not favorable for market access in general. As orlistat has been the only pharmacotherapy option on the market for around two decades, more than half of the studies included in our review evaluated the cost-effectiveness of this AOD either as the primary intervention or as a comparator. Patients in overweight or obesity who are with or without diabetes were observed in these studies. And the evaluations were conducted in various countries from different perspectives. Both cohort-based Markov model and patient-based Monte Carlo simulations were adopted in the modelling construction. Although the generalizability of these evaluations was undesirable, it is observed that the models have evolved to capture a relatively more complex disease progression course. Specifically, the early studies adopted a shorter time horizon, while the more recent studies made efforts to extrapolate the weight loss effects to the long term by incorporating the occurrence risks of complications such as type 2 diabetes and cardiovascular events. The economic evaluations focusing on LIRA 3.0mg, NB ER and PHN/TPM ER were relatively insufficient, which makes comparisons across challenging. By contrast, the latest studies on the SEMA 2.4 mg sponsored by the manufacturer seem to alter this situation. Although the three evaluations included were conducted in different settings, the cost-effectiveness of this newly approved GLP-1 drug for long-term weight management based on the Core Obesity Model was consistently promising.

The five licensed AODs for long-term weight reduction identified in this study have been approved in North America, and four of them except NB ER are available in the European markets. This scenario is probably the main reason that nearly all the included economic evaluations were conducted in countries from these regions. There was one study that was carried out in Australia, where orlistat, phentermine, and liraglutide are officially available for weight reduction. No pharmacoeconomic evidence was generated from a Chinese setting, as orlistat has been the only approved pharmaceutical option for weight reduction in China for a long time. The emerging novel drug targets for weight loss have attracted domestic pharmaceuticals and research teams. To facilitate the research and development of AODs, the *Technical Guiding Principles on the Clinical Trials of Weight Management Drugs* was enacted in 2021 by the Center for Drug Evaluation (CDE) of the NMPA as a move at the institutional level to combat obesity.

As modelling-based economic evaluations are relatively less time- and money-consuming, the majority of the included studies constructed a mathematical model to calculate the possible costs and health outcomes of the intervention of interest. Two evaluations were data-based (59, 60), and one of them was a typical piggyback study alongside the phase III clinical trial on Qsymia (59). State-transition decision analytical approaches including the Markov model and microsimulation were predominantly adopted in most of the studies on the AOMs approved earlier. In these models, a manageable number of the key health states and the state transitions were structured to capture the disease progression. One of the key model assumptions found in these studies was primarily about the length of weight loss decay. Studies proved the sensitivity of effect persistence in the model by assuming either a longer or shorter course of weight regain in the sensitivity analysis than in the base case scenario (51, 52, 54, 55, 58–62, 67, 69, 70). Naturally, the longer the weight loss sustained after the treatment cessation, the better benefit was observed. And in the modelling of these studies, the decay process of the weight loss effect was normally assumed linearly after the treatment cessation. Moreover, adverse events associated with the pharmacologic treatments were seldom explicitly incorporated into the models in the included studies. The Core Obesity Model was the only one that was applied in different studies on the same AOM SEMA 2.4 mg (51–53). It also follows a Markov model structure, which aims to reflect the natural disease course in a real-world setting by incorporating a series of obesity-related comorbidities including the occurrence of pre-diabetes evidenced in literature or pivotal clinical studies (76). The uncertainty of the modelling evaluations on AODs would be mitigated to a greater extent if more solid evidence could be achieved in the understanding of the weight rebound process after the discontinuation of treatment.

The major evaluation technique adopted in the included studies was cost-utility evaluation. Quality of life has been proved to be negatively associated with the BMI value, so in these CUA studies, quality-adjusted-life-years (QALY) gained per weight loss effect and disability-adjusted-life-years (DALY) were used as the surrogates of health utility. The methods employed in the estimation of health utilities include direct elicitation (55, 62, 64), indirect measurement with self-reported questionnaires such as EQ-5D and SF-12 v2 (58–61), as well as extraction of reference value from previous literature (51–54, 56, 57, 63, 65–69). Although using QALYs aims to facilitate the comparison across studies, the health utility values associated with one unit reduction in BMI were found to differ considerably in these studies. The comparability between studies on the cost-effectiveness of AODs would be improved if a more in-depth understanding of the linkage between quality of life, weight reduction effect, adverse events, and side effects could be obtained through clinical and real-world studies, and correspondingly better measurement of utility value could be performed.

All the included studies made efforts to examine the uncertainty through various sensitivity analyses, which constituted the good practice of reporting (77). Future studies could provide proper justifications on the selection of parameters or inputs as the covariates in the sensitivity analysis with evidence-based consideration of the nature of the disease and statistical significance. The transparency of the model structure, parameter values, and key assumptions in the included studies were found to be improved in more recent studies to facilitate stakeholders or decision-makers to obtain a fuller understanding of the generation of evaluation results from the models (78). On the other hand, the included studies except the recent two (51, 52) commonly were lack of explicit validation procedures to check the accuracy of the model.

Despite the effort, we managed to make, the current review still has some limitations. Firstly, as we only focused on the published studies, it is very likely that pharmacoeconomic evaluations not yet accessible to the public in any form were missed. Secondly, the heterogeneity in the methodological design of the included studies made the synthesis of information challenging. Thirdly, as inherent in the currently available evaluations, it would be difficult to make a judgment about the prediction of long-term weight loss effects and their impact on morbidity and mortality without the presence of long-term large-scale clinical trials and real-world observational studies.

## 5 Conclusions

This systematic review rendered a comprehensive and updated analysis of strengths and areas for improvement in the methodological design and quality of the pharmacoeconomic evaluations on the currently licensed drugs for chronic weight management. Recent CEA studies on the new-generation AOD licensed for long-term weight management indicated its great potential to better meet the clinical and market needs. More in-depth understanding of obesity and its natural trajectory as well as solid data on the long-term effectiveness and safety of AODs from future studies would facilitate the generation of pharmacoeconomic evidence with enhanced quality.

## Data availability statement

The original contributions presented in the study are included in the article/**Supplementary Material**. Further inquiries can be directed to the corresponding authors.

## Author contributions

YX: Data curation, Formal analysis, Investigation, Methodology, Writing – original draft, Writing – review & editing. HZ: Data curation, Formal analysis, Writing – review & editing. ZR: Investigation, Writing – review & editing. XC: Writing – review & editing. YL: Writing – review & editing. DY: Writing – review & editing. CU: Conceptualization, Writing – review & editing. HH: Conceptualization, Methodology, Supervision, Writing – review & editing.
