# Supplementary material for: Pharmacoeconomic evaluation of anti-obesity drugs for chronic weight management: a systematic review of literature
Source: Front Endocrinol (Lausanne). 2023 Nov 6;14:1254398. doi: 10.3389/fendo.2023.1254398 (PMC10658190; doi:10.3389/fendo.2023.1254398)
Supplement: Supplementary file 1 [file Table_1.docx]

**Supplementary Table S1. AODs for long-term use approved by FDA**

| **Drug** | **Product name** | **Dose and administration** | **FDA approval** | **EMEA approval** | **NMPA**  **approval** | **Percentage body weight change from baseline,**  **mean difference**  **(95% CI)** |
| --- | --- | --- | --- | --- | --- | --- |
| Orlistat | Xenical | 120 mg,  three times a day,  oral | 1999 | 1998 | 2000 | -3.16  (-3.53 to -2.78) |
| Phentermine/topiramate extended-release  (PHN/TPM ER) | Qsymia | 15 mg/92 mg,  daily,  oral | 2012 | No | No | -7.97  (-9.28 to -6.66) |
| Naltrexone/bupropion extended-release  (NB ER) | Contrave, Mysimba | 16 mg/180 mg,  twice a day,  oral | 2014 | 2015 | No | -4.11  (-5.19 to -3.02) |
| Liraglutide  (LIRA) | Saxenda | 3.0 mg,  daily  subcutaneous | 2014 | 2015 | No | -4.68  (-5.30 to -4.06) |
| Semaglutide  (SEMA) | Wegovy | 2.4 mg,  weekly,  subcutaneous | 2021 | 2021 | No | -11.41  (-12.54 to -10.27) |
