# Supplementary material for: Pharmacoeconomic evaluation of anti-obesity drugs for chronic weight management: a systematic review of literature
Source: Front Endocrinol (Lausanne). 2023 Nov 6;14:1254398. doi: 10.3389/fendo.2023.1254398 (PMC10658190; doi:10.3389/fendo.2023.1254398)
Supplement: Supplementary file 3 [file Table_3.docx]

**Supplementary TableS3 _Search Strategy**

**PubMed**

| **No.** | **Key words (subject headings or free terms)** |
| --- | --- |
| #1 | Obesity[MeSH] |
| #2 | “Weight Loss”[MeSH] |
| #3 | Overweight[MeSH] |
| #4 | obes*[Title/Abstract] OR "body mass ind*"[Title/Abstract] OR adiposity[Title/Abstract] OR overweight[Title/Abstract] OR "over weight"[Title/Abstract] OR antiobesity[Title/Abstract] OR "anti-obesity"[Title/Abstract] OR bodyweight[Title/Abstract] OR "body weight"[Title/Abstract] |
| #5 | #1 OR #2 OR #3 OR #4 |
| #6 | Orlistat[MeSH] |
| #7 | Xenical[Title/Abstract] |
| #8 | (phentermine[Title/Abstract] AND topiramate[Title/Abstract]) OR “phentermine/topiramate”[Title/Abstract] OR "phentermine-topiramate"[Title/Abstract] OR Qsymia[Title/Abstract] |
| #9 | bupropion/naltrexone[Title/Abstract] OR (amfebutamone[Title/Abstract] AND naltrexone[Title/Abstract]) OR (bupropion[Title/Abstract] AND naltrexone[Title/Abstract]) OR "bupropion-naltrexone"[Title/Abstract] OR Contrave[Title/Abstract] |
| #10 | Liraglutide[MeSH] |
| #11 | Saxenda[Title/Abstract] |
| #12 | Semaglutide[Title/Abstract] |
| #13 | Wegovy[Title/Abstract] |
| #14 | #6 OR #7 OR #8 OR #9 OR #10 OR #11 OR #12 OR #13 |
| #15 | Economics[MeSH] OR (“Economic Evaluation”[Title/Abstract) OR (“economic model”[Title/Abstract]) OR “Costs and Cost Analysis”[MeSH]) OR (“Cost Effectiveness”[MeSH]) OR (“Cost Utility Analysis”[Title/Abstract]) OR (“Cost Minimization Analysis”[Title/Abstract]) OR (“Cost Benefit”[MeSH]) OR (“Cost of Illness”[MeSH]) OR (“Cost Consequence Analysis”[Title/Abstract]) OR economic*[Title/Abstract] OR cost*[Title/Abstract] OR pharmacoeconom* [Title/Abstract] |
| #16 | #5 AND #14 AND #15 |

**EMBASE**

| **No.** | **Key words (subject headings or free terms)** |
| --- | --- |
| #1 | exp Obesity/ |
| #2 | exp Weight Loss/ |
| #3 | exp Overweight/ |
| #4 | (obes* OR "body mass ind*" OR adiposity OR overweight OR "over weight" OR antiobesity OR "anti-obesity" OR bodyweight OR "body weight").ti,ab. |
| #5 | #1 OR #2 OR #3 OR #4 |
| #6 | exp Orlistat |
| #7 | Xenical.ti,ab. |
| #8 | (“phentermine-topiramate” OR “phentermine and topiramate” OR "phentermine topiramate" OR phenterminetopiramate OR Qsymia).ti,ab. |
| #9 | (“bupropion-naltrexone” OR “bupropion and naltrexone“OR“amfebutamone and naltrexone”OR Contrave).ti,ab. |
| #10 | exp Liraglutide/ |
| #11 | Saxenda.ti,ab. |
| #12 | exp Semaglutide/ |
| #13 | Wegovy.ti,ab. |
| #14 | #6 OR #7 OR #8 OR #9 OR #10 OR #11 OR #12 OR #13 |
| #15 | ‘economics’/exp OR ‘economic evaluation'/exp OR 'economic model'/exp OR 'cost effectiveness analysis'/exp OR 'cost benefit analysis'/exp OR 'cost utility analysis'/exp OR ‘cost minimization analysis’/exp OR ‘cost consequence analysis’/exp OR economic*:ab,ti OR cost*:ab,ti OR pharmacoeconom*:ab,ti |
| #16 | #5 AND #14 AND #15 |

**CRD (DARE, NHS EED, HTA)**

| **No.** | **Key words (subject headings or free texts)** |
| --- | --- |
| #1 | (MeSH DESCRIPTOR Obesity EXPLODE ALL TREES) OR (Obes*) OR (Overweight) OR (anti obesity) OR (weight loss) |
| #2 | (Orlistat) OR (Xenical) OR (phentermine topiramate) OR (qsymia) OR (naltrexone bupropion) OR (Contrave) OR (Liraglutide) OR (Saxenda) OR (semaglutide) OR (Wegovy) |
| #3 | #1 AND #2 |

**ISPOR**

| Disease/Disorder | ALL |
| --- | --- |
| Topic | Economic Evaluation |
| Subtopic | ALL |
| Conference | ALL |
| Authors |  |
| Keyword | Obesity~ |
