# Supplementary material for: Pharmacoeconomic evaluation of anti-obesity drugs for chronic weight management: a systematic review of literature
Source: Front Endocrinol (Lausanne). 2023 Nov 6;14:1254398. doi: 10.3389/fendo.2023.1254398 (PMC10658190; doi:10.3389/fendo.2023.1254398)
Supplement: Supplementary file 4 [file Table_4.docx]

**Supplementary Table S4. Quality assessment of the included studies (full-text only)**

|  | **Item** | **Guidance for Reporting** | **Percentage of studies reporting**  **(out of 18 full-text articles)** |
| --- | --- | --- | --- |
| **TITLE** |  |  |  |
| Title | 1 | Identify the study as an economic evaluation and specify the interventions being compared | 100.00% |
| **ABSTRACT** |  |  |  |
| Abstract | 2 | Provide a structured summary that highlights context, key methods, results, and alternative analyses | 94.44% |
| **INTRODUCTION** |  |  |  |
| Background and objectives | 3 | Give the context for the study, the study question, and its practical relevance for decision making in policy or practice | 100.00% |
| **METHODS** |  |  |  |
| Health economic analysis plan | 4 | Indicate whether a health economic analysis plan was developed and where available | 5.56% |
| Study population | 5 | Describe characteristics of the study population (such as age range, demographics, socioeconomic, or clinical characteristics) | 100.00% |
| Setting and location | 6 | Provide relevant contextual information that may influence findings | 100.00% |
| Comparators | 7 | Describe the interventions or strategies being compared and why chosen. | 100.00% |
| Perspective | 8 | State the perspective(s) adopted by the study and why chosen. | 94.44% |
| Time horizon | 9 | State the time horizon for the study and why appropriate | 94.44% |
| Discount rate | 10 | Report the discount rate(s) and reason chosen | 88.89% |
| Selection of outcomes | 11 | Describe what outcomes were used as the measure(s) of benefit(s) and harm(s) | 100.00% |
| Measurement of outcomes | 12 | Describe how outcomes used to capture benefit(s) and harm(s) | 100.00% |
| Valuation of outcomes | 13 | Describe the population and methods used to measure and value outcomes | 83.33% |
| Measurement and valuation of resources and costs | 14 | Describe how costs were valued | 100.00% |
| Currency, price date, and conversion | 15 | Report the dates of the estimated resource quantities and unit costs, plus the currency and year of conversion. | 100.00% |
| Rationale and description of model | 16 | If modelling is used, describe in detail and why used. Report if the model is publicly available and where it can be accessed. | 68.75% |
| Analytics and assumptions | 17 | Describe any methods for analysing or statistically transforming data, any extrapolation methods, and approaches for validating any model used | 81.25% |
| Characterizing heterogeneity | 18 | Describe any methods used for estimating how the results of the study vary for sub-groups | 37.50% |
| Characterizing distributional effects | 19 | Describe how impacts are distributed across different individuals or adjustments made to reflect priority populations | 50.00% |
| Characterizing uncertainty | 20 | Describe methods to characterize any sources of uncertainty in the analysis | 93.75% |
| Approach to engagement with patients and others affected by the study | 21 | Describe any approaches to engage patients or service recipients, the general public, communities, or stakeholders (e.g., clinicians or payers) in the design of the study | 0.00% |
| **RESULTS** |  |  |  |
| Study parameters | 22 | Report all analytic inputs (e.g., values, ranges, references) including uncertainty or distributional assumptions. | 100.00% |
| Summary of main results | 23 | Report the mean values for the main categories of costs and outcomes of interest and summarise them in the most appropriate overall measure. | 100.00% |
| Effect of uncertainty | 24 | Describe how uncertainty about analytic judgments, inputs, or projections affect findings. Report the effect of choice of discount rate and time horizon, if applicable. | 100.00% |
| Effect of engagement with patients and others affected by the study | 25 | Report on any difference patient/service recipient, general public, community, or stakeholder involvement made to the approach or findings of the study | 6.25% |
| **DISCUSSION** |  |  |  |
| Study findings, limitations, generalizability, and current knowledge | 26 | Report key findings, limitations, ethical or equity considerations not captured, and how these could impact patients, policy, or practice. | 100.00% |
| **OTHER RELEVANT INFORMATION** |  |  |  |
| Source of funding | 27 | Describe how the study was funded and any role of the funder in the identification, design, conduct, and reporting of the analysis | 68.75% |
| Conflicts of interest | 28 | Report authors conflicts of interest according to journal or International Committee of Medical Journal Editors requirements | 87.50% |
